# Forecasting national and regional level intensive care unit bed demand during COVID-19: The case of Italy

**DOI:** 10.1371/journal.pone.0247726

**Published:** 2021-02-25

**Authors:** Simone Gitto, Carmela Di Mauro, Alessandro Ancarani, Paolo Mancuso

**Affiliations:** 1 Department of Information Engineering and Mathematics, University of Siena, Siena, Italy; 2 Management Engineering Group, DICAR, University of Catania, Catania, Italy; 3 Department of Industrial Engineering, University of Rome Tor Vergata, Rome, Italy; Italian National Research Council (CNR), ITALY

## Abstract

Given the pressure on healthcare authorities to assess whether hospital capacity allows properly responding to outbreaks such as COVID-19, there is a need for simple, data-driven methods that may provide accurate forecasts of hospital bed demand. This study applies growth models to forecast the demand for Intensive Care Unit admissions in Italy during COVID-19. We show that, with only some mild assumptions on the functional form and using short time-series, the model fits past data well and can accurately forecast demand fourteen days ahead (the mean absolute percentage error (MAPE) of the cumulative fourteen days forecasts is 7.64). The model is then applied to derive regional-level forecasts by adopting hierarchical methods that ensure the consistency between national and regional level forecasts. Predictions are compared with current hospital capacity in the different Italian regions, with the aim to evaluate the adequacy of the expansion in the number of beds implemented during the COVID-19 crisis.

## 1. Introduction

By giving rise to sudden surges in hospital admissions, pandemics place a heavy load on health systems [1]. The current COVID-19 outbreak is putting the entire world to test [2, 3], and has impacted countries such as Brazil, Italy, Spain, UK and the US more severely than others. In spite of mitigation measures adopted, both hospital infectious wards and especially intensive care units (ICU henceforth) have been overburdened in many countries [4]. Since shortage of ICU beds may engender a trade-off between saving the life of a patient over another, the ability to timely forecast the impact of the epidemic on ICU bed capacity usage is a critical component of adequate outbreak management [5]. Timely forecasts are in fact key to adjusting ICU capacity to meet demand or planning measures for the transfer of patients.

Researchers in the medical field typically use epidemic models of disease spread to gain insight into the transmission dynamics. These models hold two sources of uncertainty, the first linked to observational error and limited resolution and detail from available data, and the second stemming from uncertainty in the model structure, especially when confronting outbreaks triggered by a novel virus. Furthermore, some studies have underlined the sensitivity of key epidemiological parameters to model assumptions [6].

Several very recent studies have tackled the problem of predicting COVID-19 related health outcomes using data-driven models. Petropoulos and Makridakis predicted cumulative level of confirmed cases, deaths and recoveries at worldwide level using models from the exponential smoothing family [7]. They used data for the period from January 22th to March 11th, 2020 to produce ten-days-ahead point forecasts. Utkucan and Tezcan compare grey model, nonlinear grey Bernoulli model and fractional nonlinear grey Bernoulli model to forecast the number of confirmed case in Italy, UK and USA using a 35 days training sample (from March 19th to April 22th, 2020) [8]. Parbat and Chakraborty propose a prediction model of the total number of deaths, patients recovered, cumulative number of confirmed cases and number of daily cases of COVID19 in India using support vector regression [9]. The data are analyzed for the period from March 1^st^ to April 30^th^, 2020 (61 Days) with a training sample of about 60 days (30^th^ June). Ribeiro et al. build forecasts of cumulative confirmed cases over ten Brazilian states with three forecasting horizons (one, three, and six-days-ahead) [10]. A training sample of 34–53 days is used to compare six models (autoregressive integrated moving average, cubist regression, random forest, ridge regression, support vector regression, and stacking-ensemble learning). Chakraborty and Ghosh analyzed COVID-19 cases for India, Canada, France, South Korea, and the UK using ARIMA, wavelet-based forecasting techniques and a hybrid forecasting approach [11]. Their observation sample is based on 64–76 observations according to the country, through which they compare the fit of the proposed models on past data. Torrealba-Rodriguez et al. predict confirmed cases in Mexico using Gompertz, logistic and artificial neural network models [12]. Their sample includes 60 observations (from February 27, 2020 to May 8, 2020) to build seven-day forecasts (from May 9th to 16th). Singh et al. test the accuracy of a hybrid wavelet-ARIMA model using past 66 days data of death cases to generate forecasts 16 days ahead for five countries (Italy, Spain, France, USA, and UK) [13].

Phenomenological models (also known as growth models), which are used extensively in modelling and forecasting the diffusion of innovations, new technologies or new products in marketing and operations management [14], have recently been employed also to predict epidemics [15–18]. This class of models offers simple, data-driven methods that can be used with small samples and do not require hypotheses about epidemiological parameters. When addressing the problem of forecasting ICU demand during the initial phase of an epidemic, growth models represent a useful tool because they allow obtaining rapid, yet accurate predictions using short time-series and forecasting over a time horizon sufficient for ICU bed capacity adjustments to be implemented.

Another important issue when forecasting ICU demand lies in the fact that the spread of the disease has been highly variable within a single country. To illustrate, the Italian national health system’s capacity to match the demand for ICU beds has dramatically been put under stress especially in those areas of the country that were hit first and foremost [19], but less so in several other regions where the number of hospitalized patients has remained under control throughout the crisis. The uneven spread of the disease across the country implies the need for accurate forecasts of ICU demand not only at national but also at regional level. Further, it is critical that forecast models provide regional-level estimates that are consistent with national-level ones, in order to allow effectively planning healthcare capacity adjustments associated with a single region or the overall (national) healthcare system. This consistency requirement is especially relevant in Italy because the Italian National Healthcare System is organized on a regional basis. In such a context, running separate forecasts at regional and national level without a consolidation would generate inaccurate results. To ensure the reliability of consolidated multilevel forecasts and to improve the accuracy of the overall forecast, hierarchical forecast techniques can be adopted [20, 21].

This study applies three growth models and hierarchical forecasting methods to predict the demand of ICU beds at national and regional level in Italy during the most severe period of the COVID-19 outbreak. We show that, with only mild assumptions on the functional form, one model fits past data on bed demand well and can be used to forecast demand in the days ahead.

With respect to previous contributions, this study innovates by: i) proposing the use of Harvey model [22], which represents a novel application in the case of predicting COVID-19 health outcomes; ii) showing the suitability of the proposed model using a short training sample (22, 24, 26 or 28 observations) and a rather long (fourteen-days) period of forecast, in comparison with the more widely used logistic and Gompertz models; iii) introducing the hierarchical forecasting approach to predict COVID-19 ICU admissions in order to ensure consistency and increase accuracy of both national and regional series.

The relevance of the study lies in the provision of a decision support tool that can be straightforwardly implemented in countries that are being hit by the COVID-19 outbreak. Forecasts obtained can be compared with current ICU capacity, with the aim to estimate the need to either expand the number of beds or to organize the transfer of patients to neighbouring areas.

The article is organised as follows: Section 2 introduces the methodologies adopted, Section 3 provides a brief overview of the data used, Section 4 presents model fit and forecasts, while Section 5 concludes with a discussion of the results.

## 2. Methods

### 2.1. Growth models

The most frequently used approach in epidemiology for forecasting the evolution of an infectious disease adopts mechanistic models relying on assumptions about the transmission mechanisms of the disease in an infected population, which are embedded in the model parameters (e.g. number of infected over the total population, percentage of infected requiring intensive care, expected length of the outbreak). This approach uses different variants of the so-called SIR model (*susceptible*, *infectious*, *recovered*), which allows an exact evaluation of the epidemiological diffusion of the disease in the long term [23]. However, SIR models appear not to perfectly suit situations in which the transmission mechanisms of the disease are only partially known and real-time data are incomplete. As past experiences with the SARS and Ebola outbreaks have shown, knowledge about transmission mechanisms of new diseases may develop slowly, therefore requiring model assumptions that may turn out to be inaccurate in the first phases of the disease management. Specifically, in order to forecast the demand for hospital beds during the outbreak, SIR models make starting hypotheses concerning a number of parameters, which are then adjusted as more knowledge about the outbreak and the disease is accumulated [18, 24–26].

The experience with COVID-19 has further shown that it is difficult, if not impossible, for countries that are being hit by the disease to use previous estimates from other countries (e.g. data from China or Korea) as starting model parameters. In fact, comparability is severely hindered by the fact that the infection rate in the population and the mortality rate exhibit significant cross-country differences due to varying national public health practices, data collection and political containment decisions. This limitation may extend to comparisons of infection rates among regions within a country, depending on the degree of autonomy of regional healthcare bodies.

Past epidemics have taught that it is important to balance the need for detailed models against the limitations stemming from the parameter information they require. Knowing the growth rate of cases and when they will peak is critical for healthcare providers to organize an adequate response. For this reason, phenomenological models (also known as growth models) that use a fully data-driven approach are also used in forecasting the diffusion of an epidemic, in alternative to or in conjunction with SIR models [15]. In spite of their simplicity, growth models might produce good forecasts using small samples, even in the absence of hypotheses about the population, the rate of diffusion, and the upper limit of the infection curve [16, 17]. Furthermore, even if they have fewer parameters, these models may produce better forecasts than mechanistic ones for forecasts of 1–2 weeks [18].

Many functional forms have been used in epidemic forecasting research, as documented by other contributions in this journal. The logistic model is the baseline model. It is defined by the equation:

$$Y_{t}=\frac{\theta_{1}}{1+\theta_{2}exp(-\theta_{3}t)}+\varepsilon_{t}$$ (1)

Where *Y*_*t*_ is the cumulative level of the variable, *t* denotes time, Θ_1_, Θ_2_ and Θ_3_ are the parameters to be estimated using nonlinear least squares and ε_t_ is the error term. The curve is symmetric about its points of inflection (i.e., half the relevant population has the disease at the point of inflection).

The Gompertz curve is another widely applied model. Using the same notation as in (1), the equation is:

$$Y_{t}=\theta_{1}exp[-\theta_{2}exp(-\theta_{3}t)]+\varepsilon_{t}$$ (2)

Unlike the logistic, this curve is asymmetric about its points of inflection.

Flexible functional forms have been proposed with respect to the baseline logistic model [14, 26, 27]. Forecast accuracy has been shown to vary across different growth models. Young compared forecast accuracy over the last three data points for nine different models [26] and showed that the model proposed by Harvey was the most accurate (Eq 3) [22].

$$\text{ln}\left(y_{t}\right)=\beta_{0}+\beta_{1}\;\text{ln}\left(Y_{t-1}\right)+\beta_{2}t+\varepsilon_{t}$$ (3)

In Harvey’s model, *y*_*t*_ is the change of *Y*_*t*_ (that is *y*_*t*_ = *Y*_*t*_ − *Y*_*t-1*_), β are the parameters to be estimated and ε_t_ is the error term. Thus, this rate-of-change model does incorporate the variable time (t), allowing for flexibility in skew and, as discussed by Harvey and Young, it includes the logistic, Gompertz and modified exponential as special cases [22, 26]. The β parameters can be estimated by ordinary least squares. Analogously to the logistic form, the model is subject to a saturation level, although the limit is not imposed a priori but it is estimated from the data. Note also that Harvey model, unlike the models (1–2), includes an autoregressive term. Quite surprisingly, while the logistic and Gompertz models have been widely used in forecasting the diffusion of an epidemic [15–18], we are not aware of any application of Harvey model in this context.

### 2.2. Hierarchical forecasting

As suggested by previous studies, epidemic curves are characterized by different spatial and temporal patterns [27], with the consequence that regional forecasts may not reproduce the aggregate national level predictions. When series are disaggregated by geographical area (e.g. regions) or by categories, they form a hierarchy. The hierarchical forecasting approach provides a method to obtain consistent forecasts at all levels, i.e. ensures that the sum of regional forecasts is equal to the national forecasts. Several hierarchical forecast techniques have been proposed and two main alternative approaches can be identified: top-down and bottom-up methods (see the review in [20, 21]). We argue that the top-down approach is well-suited to the COVID-19 epidemic case, as bottom-level data of regional ICU admissions are quite noisy and are therefore challenging to model. Furthermore, Athanasopoulos et al. notes that the performance of bottom-up method deteriorates as the length of the forecasting horizon increases [20].

The top-down approach discussed by Athanasopoulos et al. is based on disaggregating the national forecasts according to forecast proportions of the regional series [20]. First, the method requires generating *h*-step-ahead forecasts for all the regional series ${\hat{y}}_{h,j}^{}$, j = 1,‥,J and the national series ${\hat{y}}_{h}^{}$. Next, the top-down approach disaggregates the national series in order to obtain modified regional forecasts as follows:

$${\tilde{y}}_{h,j}^{}=p_{h,j}\;{\hat{y}}_{h}^{}$$ (4)

Where *p*_*h*,*j*_ are the regional proportions, and are obtained as:

$$p_{h,j}=\frac{{\hat{y}}_{h,j}^{}}{\sum_{j=1}^{J}{\hat{y}}_{h,j}^{}}$$ (5)

Therefore, the regional forecasts are not used directly, but they drive the decomposition from the top to the bottom level, generating regional forecasts coherent with the national one.

### 2.3. Evaluating forecast accuracy

We are interested in comparing the forecast accuracy of models introduced in section 2.1–2.2. When choosing models, it is common practice to identify a training sample, where the training data is used to estimate any parameters of a forecasting method, and a test sample, that is used to evaluate its accuracy.

Using the same notation of the previous sections, we denote cumulative forecast with ${\hat{Y}}_{t}^{}$ and *Y*_*t*_ is the observation at time *t*. Two commonly used scale-dependent measures are the mean absolute error (MAE) and the root mean squared error (RMSE) [28]:

$$MAE=mean(\left|{\hat{Y}}_{t}^{}-Y_{t}\right|)$$ (6)

$$RMSE=\sqrt{mean{\left({\hat{Y}}_{t}^{}-Y_{t}\right)}^{2}}$$ (7)

It is possible to define percentage errors that are unit-free [28]. The most common used measure is the mean absolute percentage error (MAPE) that is defined as:

$$MAPE=100\;mean{\left(\left|\frac{{\hat{Y}}_{t}^{}-Y_{t}}{Y_{t}}\right|\right)}^{}$$ (8)

According to Lewis, MAPE values suggest accurate forecast when <10, good forecast when they are in the interval 10–20, and reasonable forecast when they are in the interval 20–50 [29]. Hyndman and Koehler suggest to use MAPE and MAE in evaluating forecast accuracy and for empirical comparisons because they are simple to explain [28].

## 3. Context under analysis

The Italian healthcare organizational context and experience with COVID-19 appears to be well suited to illustrate the benefits of the hierarchical forecasting approach. Since the 1990s, the Italian National Healthcare System has been organized on a regional base and exhibits significant geographical variations in health services provision and organization [30, 31]. A direct consequence is a significant heterogeneity both in the preventive measures (e.g. diagnosis through swab tests) and in the mitigation measures that each region has adopted to curb the outbreak. Furthermore, the large degree of autonomy in planning health care capacity has also determined a high variability in the number of ICU beds per capita available across regions.

As of June 15, the number of patients infected in Italy had reached 237,290 confirmed cases and 34,371 deaths according to data provided by the Dipartimento della Protezione Civile (http://www.protezionecivile.gov.it/attivita-rischi/rischio-sanitario/emergenze/coronavirus), making Italy one of the most severely affected countries by the virus. After the start of the infection in Italy in late February, the Italian Government was among the first to implement extraordinary community containment measures on March 8, in the attempt to limit the spread of contagion. However, during the months of March and April the hospital system was under heavy stress. This was especially true for ICUs, which accounted for about 14% of patients admitted to hospitals. During this critical period, due to the heterogeneous diffusion of the virus across the country, ICU demand in some regions stayed well below ICU bed capacity (e.g. in Southern regions), while in other regions capacity saturated quickly (e.g. Lombardy). Therefore, the epidemic put ICUs to test in an asymmetric manner, with some regions experiencing a sudden shortage of beds, equipment, and medical staff to respond to the COVID-19 emergency.

The healthcare system response was a significant increase in ICU capacity all over the country. Official statistics indicate that total capacity in Italy at the start of the outbreak was approximately 5,200 ICU beds, which has now been augmented to about 8,500 beds according to Bank of Italy sources [32]. However, the uniform increase in capacity in the entire national territory, coupled with the highly variable infection rates in the different regions, has led to excess capacity. We believe that the absence of accurate forecasts at regional level may have played a significant role in leading to public investment in capacity increase, which in some cases may have resulted in unneeded public expenditure.

## 4. Data

During the COVID-19 healthcare crisis, there was an immediate need to understand whether the capacity of ICU beds was sufficient to cover the rapidly rising demand. Below, we offer evidence that simple data-driven estimations were able to provide accurate early forecasts that would have been useful to support healthcare managers and policymakers.

Official statistics for Italy provide information on the total number of hospitalized patients and of ICU patients (https://github.com/pcm-dpc/COVID-19). However, given that the bed shortage was most severe for ICU and that this type of hospital department captures a high consumption of resources, the analysis was restricted to officially published data concerning net daily admissions (admissions minus discharges) to ICU with a COVID-19 diagnosis, by region. Admissions were monitored starting from February 25 (Day 1 of the outbreak) until April 3 (Day 39 of the outbreak). We consider this time interval significant, because it includes the peak of the daily admissions to ICU. After April 4 the daily variation of ICU admissions becomes negative at national level and demand pressure on hospitals reduces significantly.

Within this period, we identified a training sample composed by 22, 24, 26 or 28 observations (from February 25 to March 16, 18, 20 or 22), while using the remaining data as test sample. This choice of training sample allows testing the ability of our model to make accurate predictions with a short time-period. Additionally, it allows testing the goodness of fit of the model in the initial period of the outbreak, when there was high uncertainty concerning the success of containment measures introduced by the Italian government on March 8.

## 5. Results

**Fig 1:**
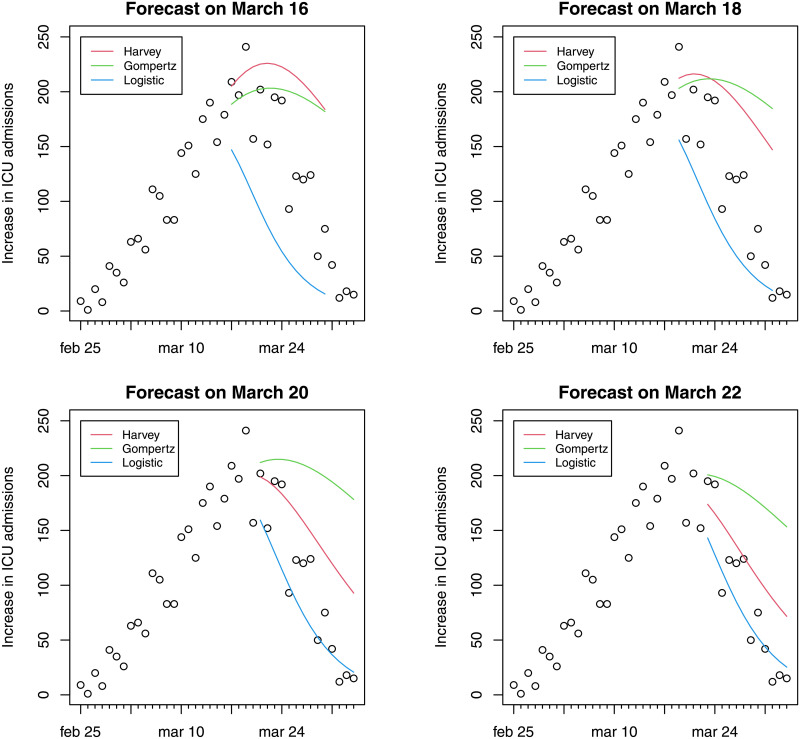
Evidence of fourteen-day forecast on daily variations (Feb 25–April 3).

**Fig 2:**
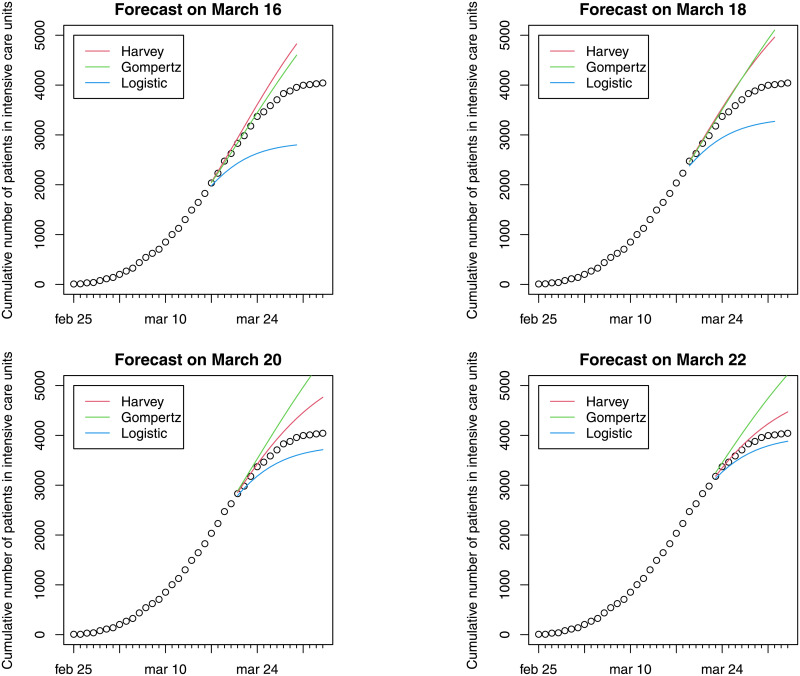
Evidence of fourteen-day forecast on cumulative ICU admissions (Feb 25–April 3).

This section presents evidence of model fit and forecasts of ICU bed demand using the methods described in Section 2: growth models and hierarchical forecasting [20]. Figs 1 and 2 report daily variations and cumulative ICU admissions at national level estimated through three different models. Fourteen-day forecasts of hospital ICU at different dates (March 16, 18, 20 or 22) are presented. The colored curves refer to forecasts made on March 16, on March 18, on March 20 and on March 22. Harvey growth model satisfactorily fits the ICU admission data for the periods considered. The model correctly predicts admissions fourteen-days ahead, although its performance worsens for forecasts made on March 22 when the net increase in ICU admissions was close to zero. In particular, Harvey growth model is able to capture the (a posteriori) evidence that the peak in the increase of daily admissions was attained during the period March 17–21. It is important to underline that even if the peak occurs during the test period, the curves reported in Figs 1 and 2 represent forecasts using the previous data and not fitted values. The forecasts based on the logistic model exhibit underestimation in the test sample, as a consequence of the low flexibility of this model. Conversely, Gompertz model shows a good fit for the forecasts made on March 16, but a steady overestimation in the remaining period.

In order to evaluate the accuracy of these forecasts, the mean absolute error (MAE), the mean absolute percentage error (MAPE) and the root mean squared error (RMSE) [29] considering the cumulative curve, are reported in Table 1. To allow evaluating the accuracy of the predictions in addition to fourteen-day forecasts, Table 1 reports three-day and seven-day forecasts for comparison. The average of these performance measures of the forecasts based on March 16, 18, 20 and 22 is also reported. These measures confirm the comparisons between models reported in Figs 1 and 2; and they show that the Harvey provides better estimates, considering forecasts on different horizons and on different days. In particular, values for MAPE suggest accurate forecasts also when the longer forecasting horizon of fourteen days is used [32]. Given the superior performance of the Harvey model as per the results in Table 1, the analysis of the estimates at regional level was carried out using only this model.

**Table 1:** Performance measures of the forecasts.

		Three-day forecast			Seven-day forecast			Fourteen-day forecast		
MAE	Forecast on	Harvey	Logistic	Gompertz	Harvey	Logistic	Gompertz	Harvey	Logistic	Gompertz
	March 16	25.01	136.05	17.73	92.38	310.29	27.38	321.40	632.94	187.56
	March 18	42.71	127.26	30.59	123.52	256.38	100.44	372.73	459.71	386.97
	March 20	49.88	64.56	102.62	104.56	149.52	225.19	291.22	244.38	610.40
	March 22	22.62	77.86	111.32	63.52	124.94	259.30	164.29	150.28	525.21
	Average	35.06	101.43	65.57	95.99	210.28	153.07	287.41	371.83	427.54
		Harvey	Logistic	Gompertz	Harvey	Logistic	Gompertz	Harvey	Logistic	Gompertz
MAPE	March 16	1.13	5.83	0.76	3.24	11.06	0.97	8.96	18.43	5.09
	March 18	1.56	4.75	1.14	3.87	8.18	3.14	9.93	12.67	10.21
	March 20	1.64	2.11	3.38	3.03	4.34	6.52	7.51	6.45	15.76
	March 22	0.66	2.31	3.30	1.71	3.42	7.02	4.17	3.93	13.41
	Average	1.25	3.75	2.14	2.96	6.75	4.41	7.64	10.37	11.12
		Harvey	Logistic	Gompertz	Harvey	Logistic	Gompertz	Harvey	Logistic	Gompertz
RMSE	March 16	26.72	155.82	22.34	116.30	359.17	36.65	423.22	729.36	279.11
	March 18	51.59	133.08	32.58	149.67	289.12	126.01	473.91	514.70	516.53
	March 20	53.46	72.34	108.36	124.43	169.85	263.69	367.20	268.43	761.14
	March 22	27.08	82.37	122.34	77.34	135.10	300.21	213.80	158.70	636.35
	Average	39.71	110.90	71.41	116.94	238.31	181.64	369.53	417.80	548.28

**Fig 3:**
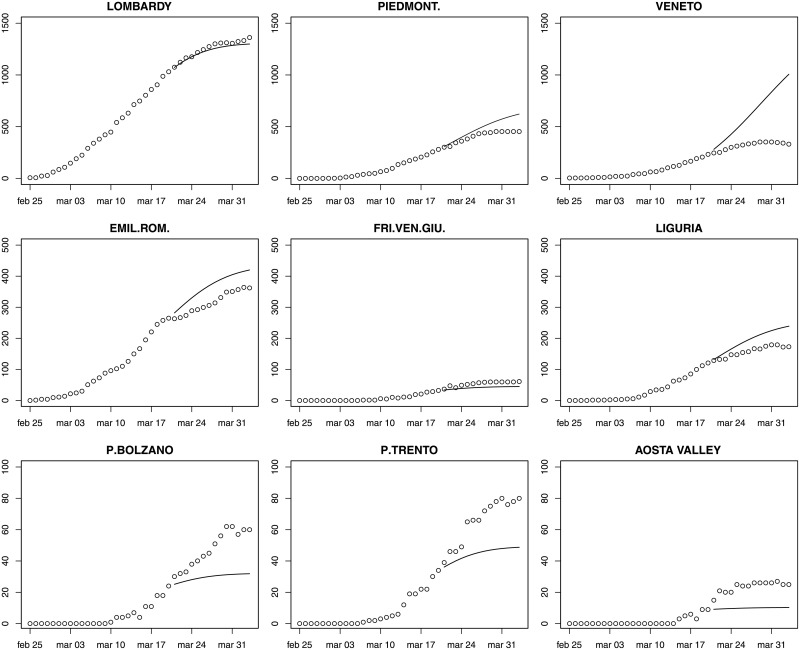
Fourteen-day forecast of ICU admissions by region. Northern Italy. Forecast on March 20.

**Fig 4:**
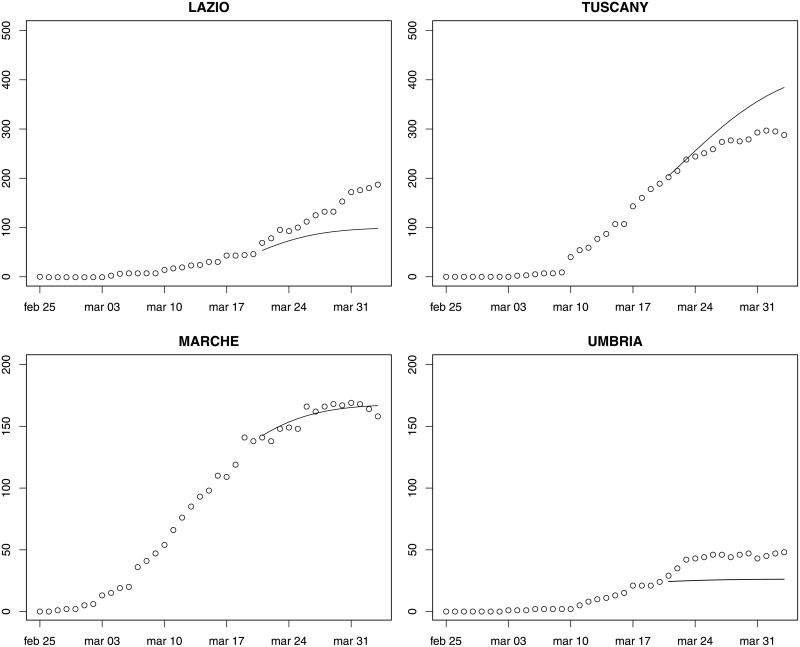
Fourteen-day forecast of ICU admissions by region. Central Italy. Forecast on March 20.

**Fig 5:**
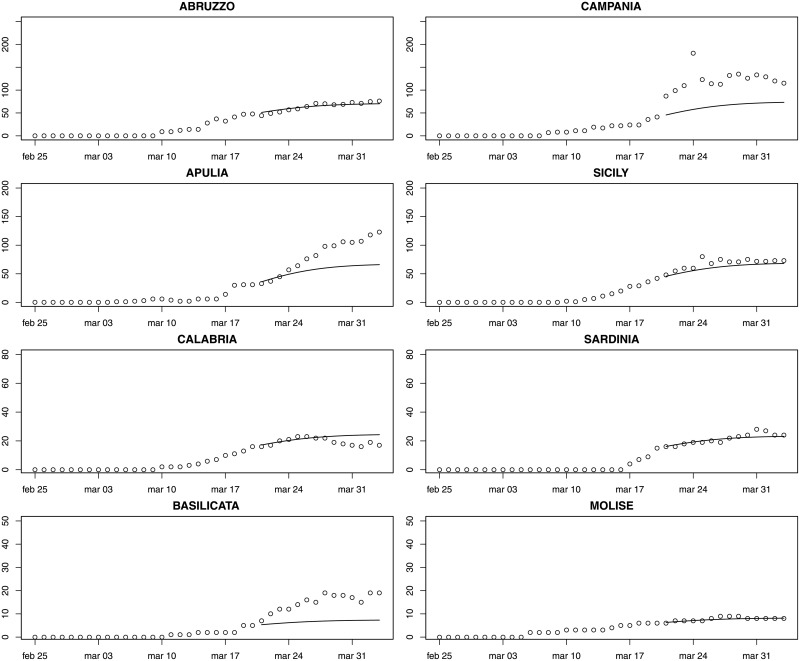
Fourteen-day forecast of ICU admissions by region. Southern Italy and main islands (Sardinia and Sicily). Forecast on March 20.

Figs 3–5 provide fourteen-day hierarchical adjusted forecasts on March 20 of cumulated ICU admissions broken down by regions, while Table 2 reports MAE, MAPE and RMSE at regional level based on Harvey model. Considering three- and seven-days forecast, MAPE values suggest accurate forecast for eight regions (<10, according to Lewis [29]), good forecast for five regions (10–20), and reasonable forecast for six regions (20–50). Two regions exhibit a MAPE greater than 50 (Aosta Valley and Campania) but their values are very close to the threshold. When forecasting over fourteen days, MAPE values suggest accurate forecast for Sardinia, Abruzzo, Molise, Marche and Lombardy, good forecast for Calabria, Piedmont, Emilia-Romagna, Tuscany and Sicily, reasonable forecast for Campania, Umbria, Bolzano, Lazio, Trento, Apulia, Friuli and Liguria, and poor forecast for Veneto, Aosta Valley and Basilicata. Valle d’Aosta and Basilicata are two very small regions that exhibited a low rate of infections, thus making forecasts very sensitive to small variations. It is noteworthy that for the three regions that were more severely affected by the epidemic (Lombardy, Piedmont, Emilia-Romagna), the forecast is either accurate or good.

**Table 2:** MAE and MAPE of the fourteen-day regional forecast (starting from March 20).

	Three-day forecast			Seven-day forecast			Fourteen-day forecast		
	MAE	MAPE	RMSE	MAE	MAPE	RMSE	MAE	MAPE	RMSE
Abruzzo	5.82	12.24	5.90	4.15	7.82	4.68	3.62	6.01	4.20
Basilicata	4.03	39.29	4.41	6.15	47.69	6.60	8.43	53.71	8.95
Calabria	1.05	6.14	1.10	1.04	5.24	1.13	3.25	18.27	4.25
Campania	48.68	49.22	49.04	61.22	50.27	66.52	58.63	47.06	61.78
Emilia-Romagna	30.4	11.29	31.72	43.87	15.21	46.21	49.82	15.69	51.35
Friuli Venezia Giulia	6.08	13.94	7.14	9.80	19.47	10.68	12.9	23.07	13.6
Lazio	20.22	24.66	20.93	24.39	24.95	25.54	45.84	32.87	52.52
Liguria	11.97	9.05	14.19	22.04	15.00	24.82	36.22	22.06	40.33
Lombardy	10.29	0.90	12.37	14.08	1.16	17.21	26.26	2.03	31.03
Marche	4.14	2.96	5.23	4.87	3.27	5.71	4.44	2.86	5.16
Molise	0.28	4.19	0.30	0.44	5.71	0.59	0.42	5.06	0.61
Bolzano P.A.	5.48	17.27	5.49	9.40	24.30	10.11	18.17	35.17	20.49
Trento P.A.	5.56	12.45	5.92	12.58	21.40	14.77	20.9	29.66	23.2
Piedmont	20.09	6.27	22.27	34.35	9.19	37.23	77.36	17.96	92.26
Apulia	2.40	6.78	2.85	10.09	15.39	13.1	27.23	28.12	33.08
Sardinia	0.60	3.70	0.78	0.88	4.83	1.13	1.42	6.16	2.04
Sicily	5.07	9.05	5.50	8.85	12.88	10.76	7.48	10.65	8.81
Tuscany	3.36	1.58	4.17	14.58	5.74	18.47	41.21	14.68	51.01
Umbria	10.73	28.86	11.87	15.65	37.04	16.57	17.67	40.02	18.24
Aosta Valley	9.31	48.84	9.64	11.65	53.72	12.02	13.65	57.12	13.96
Veneto	69.95	26.76	74.64	140.07	45.89	158.25	314.37	94.07	373.25

## 6. Application of results to ICU capacity adjustment in Italy

Predictions obtained can be compared with ICU capacity in the different Italian regions, with the aim to evaluate the adequacy of the expansion in the number of beds during the COVID-19 crisis. Table 3 reports ICU admissions at regional level (using real and forecast data) and available capacity (pre and post COVID-19 crisis). Comparison between columns (1) and (2) show that all regions have significantly expanded ICU capacity during the outbreak. Column (3) calculates the net availability of ICU for COVID-19 patients based on the assumption that ICU average occupation rate in 2019 was around 50%. Comparison of columns (3) and (5) shows that on April 3 capacity exceeded demand in all regions except Trento P.A. and Lombardy (column (7)), with average over-capacity of 50% in Northern Italy, 150% in Central Italy and 200% in Southern Italy. This geographical distribution of new ICU capacity has created a significant shortage in Lombardy, the region where the highest numbers of deaths were registered in the following weeks.

**Table 3:** Comparison between current capacity and forecasts.

Region	ICU beds before start of crisis	ICU beds at peak of crisis*	Net ICU beds available for COVID patients	ICU patients at March 20	ICU patients at April 3	Fourteen-day ICU demand forecast (March 20)	Available capacity minus COVID demand	Forecast total capacity (for COVID and other morbidities)	Forecast ICU capacity for COVID only	Forecast capacity minus demand
	*(1)*	*(2)*	*(3)*	*(4)*	*(5)*	*(6)*	*(7) = (3)-(5)*	*(8)*	*(9)*	*(10) = (9)-(5)*
Bolzano P.A.	37	86	67	24	60	49	7	77	56	-4
Trento P.A.	32	80	64	34	80	32	-16	55	36	-44
Emilia Romagna	449	708	483	267	364	422	119	743	485	121
Friuli Venezia Giulia	120	213	153	32	61	45	92	120	51	-10
Liguria	180	374	284	121	173	246	111	386	282	109
Lombardia	861	1299	868	1050	1381	1300	-513	1990	1495	114
Piemonte	327	827	663	280	452	628	211	910	722	270
Valle d’Aosta	10	35	30	9	25	10	5	17	11	-14
Veneto	494	825	578	236	335	1008	243	1443	1159	824
*Total North*	*2510*	*4447*	*3192*	*2053*	*2931*	*3740*	*261*	*5744*	4301	*1370*
Lazio	571	808	522	47	188	98	334	441	112	-76
Marche	115	217	159	138	158	176	1	268	202	44
Toscana	374	569	382	189	288	394	94	668	453	165
Umbria	70	105	70	24	48	26	22	70	29	-19
*Total Centre*	*1130*	*1699*	*1134*	*398*	*682*	*694*	*452*	*1447*	798	*116*
Abruzzo	123	172	110	48	76	70	34	151	80	4
Basilicata	49	73	48	5	19	7	29	36	8	-11
Calabria	146	206	133	16	17	24	116	111	27	10
Campania	335	440	272	41	115	73	157	276	83	-32
Molise	30	34	19	6	8	8	11	26	9	1
Puglia	304	531	379	31	123	66	256	250	75	-48
Sardegna	134	158	91	15	24	23	67	103	26	2
Sicilia	418	730	521	42	73	68	448	318	78	5
*Total South*	*1539*	*2344*	*1574*	*204*	*455*	*339*	*1119*	*1274*	*389*	*-66*
**TOTAL ITALY**	**5179**	**8490**	**5900**	**2655**	**4068**	**4773**	**1832**	**8466**	**5488**	**1420**

*Source of data [32].

Columns (8) and (9) report the forecasts of total ICU capacity (including COVID and non-COVID patients) and COVID-19 only capacity respectively. The figures have been created adding to the model forecasts a buffer of 15% (i.e. an occupation rate of 85% is assumed). The total number of ICU beds resulting from this calculation is very similar to the actual number of ICU beds created (8466 forecast vs. 8490 realized). However, the distribution of beds across the country and especially across the three different macro-areas of Italy (North, Centre and South) is significantly different, with forecasts from our model assigning the greatest portion of capacity increase to northern regions. Column (10) reports the difference between forecast capacity and actual ICU admissions on April 3, showing that the allocation of new capacity proposed by the model would have avoided the shortage of ICU beds experienced by Lombardy (-513 real data vs + 114 forecast). Overall, our model creates fewer shortages (-258 in our model vs -529 realized), although these are spread over several regions.

## 7. Conclusions and limitations

The first wave of the COVID-19 epidemic that has occurred in the first semester of 2020 has shown the difficulty of reliably predicting the number of patients requiring ICU hospitalization. Healthcare managers and policy makers at regional level critically need this information in order to rapidly adjust capacity or to organise the transfer of patients from one region to another.

This study has made two contributions to the problem of managing ICU capacity during the COVID-19 outbreak. The first contribution is the identification of a flexible functional form for predicting ICU admissions using past data that allows fitting short time series and correctly predicts the peak of ICU admissions. Results are especially relevant because the dates at which forecasts are made range from one to two weeks after the start of the lockdown in Italy, i.e. when there was high uncertainty on the effects of the community containment measures undertaken. This model has been compared to two of the most frequently used growth models, showing better performance. The second contribution concerns the decomposition of national-level forecasts of ICU admissions into regional-level predictions, with the aim of producing consistent and accurate forecasts at both levels. Forecasts of ICU capacity expansion substantially align with realized capacity expansion at national level. However, the decomposition proposed by our model assigns a greater portion of beds to Northern Italy, the area of the country that was ravaged by the virus, at the expense of creating some shortages in the South. Although our model forecasts would have avoided creating the major ICU capacity shortage in Lombardy that was unfortunately observed, we realize that such planning would have required centralised healthcare planning at national level, which is at odds with the current regional structure of the Italian healthcare system.

Limitations of the study include the fact that forecasts are based on three growth models while it would be of interest to compare forecasts from several different functional forms including other methods. Another limitation arises from the reliability of data concerning ICU demand, given that during the peak of the crisis some regions decided not to admit to hospitals patients whose conditions were too severe and who were unlikely to survive. Finally, the exact rate of occupancy of ICU beds for non-COVID inpatients is not available at local level, and this has limited ability to exactly assess the capacity gap. Despite these limitations, we are confident that the study can be useful to forecast ICU capacity needs, which may support healthcare decisions in countries that are currently experiencing COVID-19.
